# Bacterial Metabolites of Human Gut Microbiota Correlating with Depression

**DOI:** 10.3390/ijms21239234

**Published:** 2020-12-03

**Authors:** Olga V. Averina, Yana A. Zorkina, Roman A. Yunes, Alexey S. Kovtun, Valeriya M. Ushakova, Anna Y. Morozova, George P. Kostyuk, Valery N. Danilenko, Vladimir P. Chekhonin

**Affiliations:** 1 Vavilov Institute of General Genetics, Russion Academy of Sciences, 119991 Moscow, Russia; zorkina.ya@serbsky.ru (Y.A.Z.); kovtunas@gmail.com (A.S.K.); Valerid@vigg.ru (V.N.D.); 2 Department Basic and Applied Neurobiology, V.P. Serbsky Federal Medical Research Centre of Psychiatry and Narcology, 119034 Moscow, Russia; Ushakovavm@yandex.ru (V.M.U.); hakurate77@gmail.com (A.Y.M.); Chekhoninnew@yandex.ru (V.P.C.); 3 Mental-Health Clinic No. 1 Named after N.A. Alexeev of Moscow Healthcare Department, 117152 Moscow, Russia; Kgr@yandex.ru; 4 Department of Biology, Lomonosov Moscow State University, 119991 Moscow, Russia; 5 Faculty of Ecology, International Institute for Strategic Development of Sectoral Economics, Peoples’ Friendship University of Russia (RUDN University), 117198 Moscow, Russia; 6 Department of Medical Nanobiotechnology, Pirogov Russian National Research Medical University, 117997 Moscow, Russia

**Keywords:** gut microbiota, depression, gut-brain axis, biomarkers, neurotransmitters, aminoacids, functional foods, psychobiotics

## Abstract

Depression is a global threat to mental health that affects around 264 million people worldwide. Despite the considerable evolution in our understanding of the pathophysiology of depression, no reliable biomarkers that have contributed to objective diagnoses and clinical therapy currently exist. The discovery of the microbiota-gut-brain axis induced scientists to study the role of gut microbiota (GM) in the pathogenesis of depression. Over the last decade, many of studies were conducted in this field. The productions of metabolites and compounds with neuroactive and immunomodulatory properties among mechanisms such as the mediating effects of the GM on the brain, have been identified. This comprehensive review was focused on low molecular weight compounds implicated in depression as potential products of the GM. The other possible mechanisms of GM involvement in depression were presented, as well as changes in the composition of the microbiota of patients with depression. In conclusion, the therapeutic potential of functional foods and psychobiotics in relieving depression were considered. The described biomarkers associated with GM could potentially enhance the diagnostic criteria for depressive disorders in clinical practice and represent a potential future diagnostic tool based on metagenomic technologies for assessing the development of depressive disorders.

## 1. Introduction

Depression is currently ranked fourth among the causes of disability worldwide [1]. Every fifth person in developed countries has been subject to a depressive disorder at some point, and the situation is even worse in third world countries. Depression is the most common type of psychiatric disorders today, making antidepressants some of the most commonly prescribed drugs [2,3]. According to the diagnostic and Statistical Manual of Mental disorders (DSM-5), major depressive disorder (MDD) is diagnosed sustaining the following symptoms for at least two weeks: depressed mood, excessive guilt, anhedonia, suicidal ideation, changes in appetite and sleep, psychomotor retardation, poor concentration, and fatigue. MDD is a complex and heterogeneous psychiatric disorder and no reliable biomarkers currently exist that have contributed to objective diagnoses and clinical therapy [4].

The known mechanisms of origin, development, and maintenance of a depressive state are multifactorial and are determined by polymodal changes in the metabolic, immune, endocrine, gastrointestinal, and central nervous systems (CNS) [5]. The gastrointestinal tract with its microbiota interacting with external environmental signals, stress, nutrients, internal systems, including the brain, may be involved in the development of depressive states too [6]. There is ample evidence for an association between the GM and the pathophysiology of depression [7]. Different stress (social or emotional, chemical, physical, a poor diet, etc.) have the potential to alter the taxonomic composition of bacterial communities in the gut, and, as a result, leads to changes in various metabolic pathways [8]. This leads to a systematic inflammatory process, which covers the other systems and organs of humans. Over the last decade, many literature reviews have described the relationship between the gut microbiome and depression, each from a slightly different perspective [9,10,11,12]. At the current level of research, the most important point is to identify various metabolite biomarkers that correlate with depressive states. Metabolic syndrome is well-documented in MDD patients and 1.5 times higher than in the non-depressed population [13]. Isolating those that can be determined directly or indirectly by the microbiome is of great importance, both for creating diagnostic systems for identifying depression as a disease, and for choosing strategies aimed at restoring the normal functioning of the microbiota and, through it, restoring human mental health.

This review examines various low-molecular compounds as potential biomarkers of depression in correlation with the metabolism of the GM, as well as ways to correct the microbiota.

## 2. Role of the Gut Microbiome in the Gut-Brain Axis

The gut microbiota is conceptualized as a virtual organ playing a key role in maintaining homeostasis and health in humans. The GM consists predominantly of bacteria which outnumber archaea, fungi, and viruses inhabiting the intestines by a ratio of 10:1 [14]. The collective genetic makeup of the GM contains 150 times as many genes as the human genome, thereby expanding the natural capacity of humans [15]. Numerous current studies of the GM revealed its active involvement in the bidirectional communication between the gut and the brain. The discovery of the microbiota-gut-brain axis (MGBA) induced scientists to study the role of the GM to neurological health [16,17,18]. Clinical and experimental findings are evident of the interactions occurring locally between the GM and the intestinal cells or the enteric nervous system (ENS), and also between this microbial community and the brain via neuroendocrine and metabolic pathways. The human GM is capable of producing hundreds of metabolites that directly affect most systems and organs, including the intestinal epithelium and the enteric nervous system, and also the brain [19].

The role of the intestinal symbionts in the development and function of the brain was studied in different animal models [20]. The obtained data demonstrate the enormous effect the commensals have on a wide range of behavioral aspects, including social behavior, mood, and anxiety. MGBA covers a wide range of immune and endocrine functions [21,22] and is a key player in the initial stage of central nervous system (CNS) development in humans [23]. Disturbances in the harmonious interactions between the microbiota and its host during critical stages of child’s development can cause profound damage to gut-brain signaling pathways and put an individual at risk for psychiatric disorders later in life, including depression [16,24]. For instance, among the factors contributing to brain diseases is early life exposure to harmful food derivatives and small molecules derived from microbial metabolism, which first permeate into the bloodstream and then to the brain [25]. Thus, the identification of damaging early life events can help establish the causes of depression while diagnosing a patient. These include the exposure to antibiotics, unbalanced nutrition, stress, and other factors [26].

**Figure 1:**
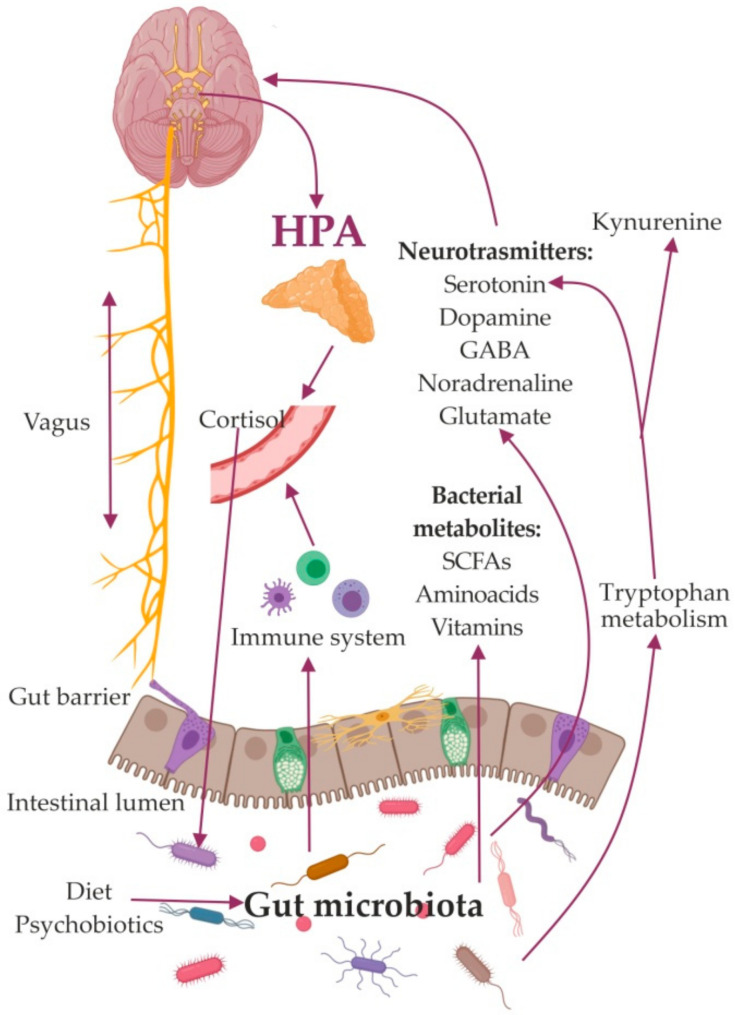
Schematic image of the role of the gut microbiome in the gut-brain axis.

So far, a few major mechanisms mediating the effects of the GM on the brain have been identified. Among these are the activation of the vagus nerve [27,28,29] and the immune system [30,31], production of metabolites, and compounds with neuroactive properties [32,33]. Bacterial products participate in stimulation of central receptors, peripheral stimulation of neural, endocrine, and immune mediators, and epigenetic regulation of histone acetylation and DNA methylation, which are implicated in depression. (Figure 1).

The mechanisms found to be linked to depression also include a dysfunctional hypothalamic–pituitary–adrenal (HPA) axis [34]; immune-inflammatory, oxidative pathways [35]; altered vagus nerve tone [36]; region-specific changes in brain-derived neurotrophic factor concentrations [37]; and an imbalance between neural excitation and inhibitory signaling [38].

Gut microbes may trigger neurotransmitter release via Toll-Like Receptor (TLR) signaling on epithelial, immune, and neuronal cells [39,40]. Bacteria can synthesize neuromodulators, stimulated specific epithelial cells of the intestines, to secrete molecules responsible for signal transmission through the enteric nervous system [31,32]. Using their own signaling pathways, they regulate the release of neurotransmitters from enterochromaffin cells [41,42,43,44]. Bacteria are called vectors because they can deliver neuroactive compounds to the receptors of epithelial cells. These bacteria-derived neuroactive compounds can play a significant role in modulating the signals of the GM caused by changes in concentration of ions (K^+^, N^+^, Ca^2+^, Cl^−^, etc.), as well as exogenous agonists and antagonists coming from food. Thus, bacterial neuroactive substances can control depolarization in the synaptic area of neurons containing neurotransmitters. Bacteria can produce the neurotransmitters GABA, serotonin, dopamine, and acetylcholine [44,45], which can affect the emotional state by binding to specific receptors on nerve and immune cells in the central and peripheral nervous systems [8].

Elucidating these mechanisms is crucial for understanding the etiology of depression and developing new strategies aimed at harnessing the beneficial psychotropic effects of these molecules. It is also important to note that not all bacterial metabolites are beneficial. Some metabolites are rather harmful and conducive to depression.

## 3. The Gut Microbiome of Patients with Depression

A comprehensive literature analysis of human studies on the gut microbiome and depression showed significant differences between the GM of patients with depression and healthy controls [46,47]. Overall, the distinctive characteristic of the GM in patients with depression is a decline in richness and alpha- and beta-diversity [48,49]. In depressed patients, decreased microbial diversity was found in most studies. At the phylum level, there were inconsistencies in the abundance of *Firmicutes*, *Bacteroidetes*, and *Proteobacteria.* However, high abundance in *Actinobacteria* and *Fusobacteria* phyla were observed in people with depression [46]. Naseribafrouei et al. detected low abundance of the order *Bacteroidales* and a high abundance of the *Lachnospiraceae*, and *Prevotellaceae* families and the *Prevotella* species [48,50]. Higher levels of *Bacteroidetes*, *Proteobacteria,* and *Acinobacteria*, and lower levels of *Firmicutes* were also reported [48]. In the study of Liu et al. and Aizawa et al. the GM of patients with depression contained lower levels of *Lactobacillus*, *Bifidobacterium*, *Firmicutes*, *Faecalibacterium,* and *Ruminococcus* and higher levels of *Prevotella*, *Bacteroides,* and *Proteobacteria* [51,52]. *Flavonifractor* was reported to be increased in MDD patients [48] and *Coprococcus* and *Dialister* were consistently depleted [53]. Szczesniak et al. found that *Faecalibacterium*, *Alistipes,* and *Ruminococcus* correlated with depression [54]. Altogether, the recurrent pattern seen in depression is an overabundance of potentially harmful and inflammatory bacteria such as *Actinobacteria* and *Enterobacteriaceae* combined with a decrease in beneficial bacteria such as *Faecalibacterium* and *Firmicutes* in general [48,50,55,56]. Although all these studies focused on finding the distinctive features of the GM in depression, the constantly arising contradictions between them led to lack of consensus. It was argued that the contradictions are due to inconsistencies in the methodology between the studies such as the diagnostic criteria, grouping criteria, detection methods of fecal microbiota, etc. Hence, some of the suggested solutions to this problem include shifting from 16SrRNA sequencing to whole metagenome sequencing, which allows focusing on the functional capacity of the GM, a more significant indicator of its potential [57]. In addition, consistent demographic and clinical characteristics of recruited subjects are needed. The gut is a complicated ecosystem, which can be influenced by various factors such as, genetics [58], diet [59], age [60], and regional variations [61].

Today, scientists acknowledge that the GM is an organ fine-tuned to the pathological processes in the organism as exemplified by depression. The GM produces a wide array of metabolites, some of which correlate with symptoms of depression [62,63]. Some of these metabolites exhibit neuroactive properties and are involved in immunomodulation. Table 1 summarizes the bacterial enzymes involved in the production of many metabolites relevant to depression and Table 2 provides a summary of studies of altered metabolites in patients with depression. Studying the influence of the GM on the biomarkers of depression will greatly improve our understanding of its pathogenesis and our chances of diagnosing it accurately in the early stages before it escalates into a full-blown condition. In the following sections, we contemplate the bacterial metabolites that are potentially involved in depression.

## 4. Neurotransmitters

Neurotransmitter imbalance is an almost inseparable feature of depression. The monoaminergic neurotransmitter deficiency hypothesis posits that symptoms of depression arise from insufficient levels of the monoamine neurotransmitters serotonin, noradrenaline, and/or dopamine. Production and release of neurotransmitters are among the numerous functions executed by the GM [107].

### 4.1. Serotonin

Serotonin (5-HT) is a neurotransmitter essential for the generation of emotions. In depression, however, the metabolism and reuptake of 5-HT from the synaptic cleft no longer functions properly. A disrupted serotonergic system has been often cited as one of the causes of depression, impulsivity, and in some cases, suicide. Today, the central role that 5-HT plays in the pathophysiology of depression raises little doubt. This concept is supported by clinical evidence from people affected with depression whose symptoms worsened when their serotonergic system was impaired [108]. Although plasma 5-HT levels do not correlate with 5-HT levels in the brain [109], it is still sensible to study 5-HT in the gut as a biomarker of depression.

Over 90% of the total amount of 5-HT in the human body is produced in the gut [43,110]. One way microbes alter 5-HT levels in the gut is by secreting butyrate, which is known to stimulate 5-HT synthesis in intestinal enterochromaffin cells (ECs) [43,110]. The production of 5-HT was also reported [67] by *Candida* spp., *Streptococcus* spp., *Escherichia* spp. and *Enterococcus* spp. [68] *Hafnia alvei*, *Klebsiella pneumonia*, *Lactobacillus plantarum,* and *Morganella morganii*, and others [64,65,66]. Two metabolic pathways of 5-HT have been identified in bacteria based on whether they are more similar to plants (decarboxylation of TRP to tryptamine followed by hydroxylation) or animals (hydroxylation to 5-hydroxytryptophan and then decarboxylation). The animal-like pathway was more prevalent in the microbial genomes. Although, experimental studies have demonstrated the ability to produce 5-HT for approximately 10 genera of gut-dwelling microbes, the presence of these pathways was only confirmed in half of these genera. At the same time, some genera, such as *Akkermansia*, *Alistipes,* and *Roseburia* were only predicted to be able to produce 5-HT based on their genome analysis [53]. The GM can also have a significant impact on 5-HT levels in the body by sequestering tryptophan, the precursor of 5-HT, and converting it to tryptamine, thereby denying the brain its supply of tryptophan, which is much needed for 5-HT production [107,111]. Sterile mice exhibit a significant reduction in 5-HT levels in both plasma and the brain compared to control animals [43]. There is also evidence that the recolonization of the large intestine of germ-free (GF) animals normalizes their 5-HT levels [112].

Despite the fact that depression is mainly treated by serotonin-altering drugs, there is no exhaustive explanation of the underlying mechanism at work. Meanwhile, bacteria have emerged as a new target for depression, as more evidence points to their ability to regulate 5-HT levels in the body.

### 4.2. Catecholamines

Depression has been repeatedly linked repeatedly changes in the catecholamine profile of the body. L-phenylanalnine, the precursor of the main catecholamines, adrenaline, noradrenaline and dopamine, is associated with depression [113]. A study by Chen et al. demonstrated recently impaired metabolism of L-phenylalanine in middle-aged patients with depression [114]. The view of L-phenylalanine metabolism as a biomarker of depression is also supported by animal studies. For instance, rats displaying a depressive-like phenotype as a result of exposure to chronic and mild unpredictable stress were also characterized by lower levels of L-phenylalanine [98,99]. It should perhaps be noted that L-phenylalanine was one of the metabolites used to differentiate responders from nonresponders to medication [115]. Biosynthetic pathways of L-phenylalanine in bacteria were described in *Corynebacterium glutamicum* and *Escherichia coli* in the review of Ikeda [73].

Furthermore, individuals with bipolar and unipolar depression were marked by increased levels of noradrenaline in the blood plasma and urine [95]. Many metabolomic analyses have also reported altered dopamine levels in depression. For instance, Pan et al. asserted that dopamine plasma levels in patients with depression were higher [93], while the results of Zheng et al. reported lower dopamine levels in peripheral blood cells [94]. Dopamine is the primary neurotransmitter in the reward pathway [116] and the driver of motivated behavior. It is also involved in motor functions and the regulation of cognitive processes [117]. Aberrant functioning of the dopaminergic system is associated with anhedonia, one of the key symptoms of depression [116]. Moreover, depression is characterized by an underactive dopaminergic system. This could be due to weak binding of the dopamine transporter as concluded in a study of the brains of patients with depression using a PET scan [118]. Further evidence bringing the dopaminergic system and depression closer together comes from interventional studies. While pharmacological treatment aimed at blocking or lowering dopamine levels aggravate the symptoms of depression [119], some dopamine agonists can mimic the effects of antidepressants [120]. Moreover, some antidepressants, such as buprapion and venfloxacin, possess dopamine reuptake activity [119,121], and there is a correlation between the positive effects of transcranial magnetic stimulation and blood levels of homovanillic acid—the main metabolite of dopamine [122].

Animal studies also show a clear link between the dysfunction of the dopamine system and depressive-like behavior [123]. For example, stress-mediated changes in the hippocampus and PFC of rodents have been suggested to reduce the release of dopamine and induce anhedonia. D-2 receptor agonists exhibit therapeutic effects in animals. Chronic treatment with quinpirole and bromocriptine mimicked the behavioral effects of antidepressants in the sucrose consumption model [124].

Since the GM regulates the activity of many brain regions, including those related to the mesocorticolimbic system [125], it is well-founded that the dopamine system does not function independently from the GM. Such communication occurs via the hypothalamic-pituitary-adrenal axis [126], the immune system [127], and the vagus nerve [128,129]. Stimulation of the vagus nerve in the intestinal tract increases the release of dopamine in the brain [130]. Not only do some members of the GM affect the host organism by synthesizing catecholamines, but by doing so, they regulate the growth of other bacteria as well [107]. Dopamine, for instance, is produced by *Bacillus*, *Serratia*, *Lactobacillus*, *Klebsiella*, *Morganella* [45], and *Escherichia coli* [64]. In addition, the growth of enterohemorrhagic *E. coli* O157: H7 is stimulated by noradrenaline and dopamine [131].

Thus, since the influence of symbiotic microorganisms on the dopamine system is obvious, and based on the latter being considered one of the organic manifestations of depression, we can draw a line between the GM and depression.

### 4.3. GABA

One of the neurotransmitters robustly associated with anxiety and depression besides 5-HT and dopamine, is gamma aminobutyric acid (GABA), the major inhibitory neurotransmitter in the mammalian CNS. GABA levels were found to be decreased in the blood serum of about one-third of patients with MDD, as well as in patients with mania and bipolar patients [53].

GABA owes its sedative action to its ability to bring a nerve impulse to a halt by binding to GABAergic receptors in the CNS. Transmembrane GABAergic receptors are located in the chemical synapses throughout the autonomic and central branches of the nervous system and in various tissues and organs of the human body (intestines, stomach, pancreas, kidneys, lungs, liver, and others). GABA_B_ receptors receive special attention in depression as they were recognized as one of the main targets of the disease [132]. For instance, benzodiazepines, which are prominently known to increase the affinity between GABA and its receptors, have been widely used as antidepressants. Animal studies show that a decrease in GABA_B_ receptors is associated with depressive-like behavior. Nevertheless, the use of GABA_B_ antagonists rather than the agonist baclofen was proven more effective for treating depression [133,134]. The absence of GABA_B_ receptors, as demonstrated in knockout mice, has been shown to have favorable effects [135]. Inconsistency in this field could arise from the fact that the two isoforms of GABA_B_ receptors sometimes elicit contradictory results. The implication of GABA_B_ receptors in depression is strongly believed to be related to their role in influencing the serotoninergic pathway [136]. Most cell bodies of neurons in the raphe nuclei express GABA_B_ receptors and were shown to react to baclofen by increasing their fire rate and thereby the release of 5-HT. Intake of *L. rhamnosus* altered the central mRNA expression of GABA_A_ and GABA_B_ receptors while reducing depressive and anxiety-like behavior in mice. The major contribution of this study was that it elegantly proved that *L. rhamnosus* produced the mentioned effects via the vagus nerve [129].

The data from several studies suggest that the GM does contribute to blood GABA levels. For instance, in mice with a disrupted GM, one of the many altered metabolites in the blood in consequence was GABA [137]. It is known that bacteria, such as *Lactobacillus* and *Bifidobacterium,* can synthesize GABA from dietary glutamate. *Lactobacillus rhamnosus* has been shown to reduce anxiety and depressive behavior while increasing GABA levels in the hippocampus [49]. *Bacteroides* spp. produce large quantities of GABA. Transcriptome analysis of stool from healthy individuals showed that the pathways of GABA production are actively expressed by *Bacteroides*, *Parabacteroides*, and *Escherichia* species. A recent study combining 16S rRNA sequencing with fMRI imaging, found a negative correlation between the abundance of fecal Bacteroides and the default mode network in the brains of patient with MDD [71]. Interestingly, *Bacteroides* spp., and to a lesser extent *Parabacteroides* spp., produce GABA within the near-neutral pH range of the human large intestines. In silico analysis predicted that at least 97 microorganisms possessed the ability to produce GABA, mostly using the enzyme glutamate decarboxylase. More than 25% belonged either to the genera *Bacteroides* or *Parabacteroides*. Moreover, the study identified 102 microorganisms that potentially consume GABA as a source of energy. The majority of those species belonged to the genera *Pseudomonas*, *Acinetobacter*, and *Mycobacterium* [71].

Bacteria produce gamma-aminobutyric acid via the enzyme glutamate decarboxylase, which irreversibly decarboxylates l-glutamic acid [138]. The gene encoding the enzyme glutamate decarboxylase, *gadB,* was identified in the genomes of many bacterial species, including those of bifidobacteria and lactobacilli. After decarboxylating l-glutamic acid, the *gadB* gene requires an antiporter, encoded by *gadC* and usually located in its proximity, to excrete GABA through the bacterial wall [70].

In the intestinal tract, microbial-derived GABA can trigger the release of other neurotransmitters from epithelial cells [42]. GABA also regulates the expression of proinflammatory interleukins by binding to various types of immune cells expressing GABA receptors, which makes it involved in immunomodulation [139]. Based on ligand-based in vivo studies, it has been inferred that GABA in the gut acts as a regulator of secretory and motor activities with an anti-inflammatory effect [139]. Microbe-derived GABA can cross the intestinal barrier via H+/GABA symporter [140] and subsequently interact with GABA receptors and transporters that are widely expressed on enteric neurons and vagus afferents [141].

### 4.4. Glutamate-Glutamine Cycle

Glutamate (GLU) is the primary and most abundant excitatory neurotransmitter in the CNS. Glutamatergic activity in the brain is dictated by the degradation of glutamine to GLU as a part of the glutamate-glutamine cycle. Thus, the glutamate-glutamine cycle is crucial for normal glutaminergic neurotransmission. A dysregulated glutamatergic system is viewed as one of the biological underpinnings of depression [142].

The role of GLU is central to the neuroplasticity hypothesis of depression [143]. Many animal models are grounded on the premise that depression could be explained in terms of disturbances of brain glutamatergic pathways [144,145]. Zhang et al. reported that the expression of the excitatory amino acid transporter 2 (EAAT2), the major glutamate transporter in the mammalian brain was upregulated, which is consistent with studies of postmortal brain samples from patients with depression [142,146] and the biological basis of the learned helplessness theory of depression [147]. EAAT2 is a key membrane transporter in glial cells that plays an important role in the glutamate/glutamine cycle [148], which is indispensable for the communication between glia and neurons [149]. In patients with depression, GLU and glutamine levels in the cerebrospinal fluid are increased compared to healthy controls [96]. Moreover, the therapeutic effects of cytidine in patients with bipolar depression were ascribed to its ability to decrease glutamine/glutamate levels in the brain [150]. A prior meta-analysis of 12 association studies had reported increased plasma GLU levels in patients with MDD [97].

There could be an indirect link between glutamatergic transmission in the ENS and depression. The high incidences of inflammatory bowel syndrome among patients with depression imply that depression could originate in the gut [151]. Thus, disruption of glutamatergic transmission in the ENS, a major regulator of motor and secretory regulator, could be one of the factors accounting for inflammatory bowel syndrome. A large-scale metagenomic-wide association study of 1070 individuals affected with depression revealed that depleted GLU degradation genes in the microbiota were associated with depression [53].

Bacteria can alter the GLU luminal and plasma levels in many ways. Most bacteria consume GLU and use it in the Krebs cycles to generate energy. Besides consuming it, some synthesize GLU as a by-product of fermentation [152]. Moreover, the production of GLU is not restricted to prokaryotic cells but extends to epithelial cells as well. Based on this information, we can conclude that GLU is a common currency in the mammalian body. The GLU-consuming bacterium *B. thetaiotaomicron*, reduced GLU levels in both plasma and the gut [74]. Unsurprisingly, a correlation has been found between circulating GLU and glutamine plasma levels and certain bacteria of the genus *Ruminococcus* possessing the necessary genetic machinery to convert glutamine to GLU [74]. Moreover, *Corynebacterium glutamicum*, *Brevibacterium lactofermentum,* and *Brevibacterium avium,* convert L-glutamate into D-glutamate, and use it as a building block of their peptidoglycan cell wall. Bacteria carry out this reaction using the glutamate racemase MurI [153]. *Bacteroides vulgatus* and *Campylobacter jejuni* were shown to affect GLU metabolism by decreasing 2-keto-glutaramic acid levels. *L. rhamnosus* produced GABA and GLU in vitro using glutamate decarboxylase and glutaminase, respectively [72,74]. These biosynthetic machineries utilized by microbes to synthesize GLU and GABA are identical in neurons [154], which support the interkingdom communication of microbial GABA [155].

One of the main discoveries in understanding the mechanism of depression is the identification of dysfunction in neurotransmission. Gut bacteria directly or indirectly can affect the content of serotonin, catecholamines, GABA, etc., and modulate the functioning of neurotransmitter systems, taking a possible role in the pathogenesis of depression. Therefore, such bacteria as biomarkers of depressive disorders can be used in clinical practice as part of the complex diagnostic systems for the analysis of the GM.

## 5. Amino Acids as Biomarkers of Depression

Gut bacteria play an important role in host amino acid (AA) homeostasis through multiple pathways. Metabolic interactions of AAs exist across a large number of gut bacterial species. Such interactions occur between bacteria and the host either at the local level (mucosal cells), systemic level (whole body) or even within different compartments of the intestine. Gut bacteria devised multiple strategies to survive in the intestine by modulating their AA metabolic pathways. Studies over the last decades have shown that gut bacteria can affect the physiology and metabolism of their neighboring species and eukaryotic host through the synthesis of a wide variety of AAs and their metabolites.

Since an aberration of AA metabolism has been associated with depression, many studies focused on finding the concrete AAs that could be used as biomarkers. Table 3 presents an overview of studies focusing on AA levels in animals and patients. According to a clinical study, blood levels of serine, methionine, asparagine, glutamine, and tryptophan (TRP) were decreased in patients with depression [156]. Another study determined that patients with depression had elevated plasma levels of phenylalanine, aspartate, serine, and γ-glutamyl AAs (γ-glutamyl leucine and γ-glutamyl glutamine) [113,149]. AA metabolites involved in the synthesis of branched-chain AAs, namely leucine, isoleucine, threonine and methionine, were also found to be dysregulated in patients with MDD [157].

Animal studies demonstrated that AA blood levels are influenced by the GM and are linked to depression, too. Analysis of the fecal metabolome of rats exhibiting depressive-like behavior revealed changes in the levels of the AAs L-threonine, isoleucine, alanine, serine, tyrosine, and oxidized proline. Interestingly, changes in plasma AA levels correlated with both the phylogenetic composition and changes in the AA levels observed in the fecal metabolome. A conclusion can be drawn, tying AA metabolism in the GM to an altered AA profile in the blood circulation, which can lead to behavioral changes in the characteristics of depression [158].

Metabolites of arginine catabolism are considered relevant to depression. Many studies highlighted the antidepressant and anxiolytic effects of putrescine and agmatine, a naturally occurring chemical through decarboxylation of arginine. Table 4 highlights the studies where the antidepressant and anxiolytic effects of AAs were shown. These AAs are likely to act as inhibitors of nitric oxide synthase and blockers of *N*-methyl-d-aspartate receptors, which make them promising candidates for future drugs. [159]. Decreased l-arginine levels in depressed patients could account for the decrease in NO metabolites [160]. In the study carried out by Lu et al. decreased plasma levels of aspartate, GLU and GABA, as well as increased levels of NO, were suggested as biomarkers of a melancholic form of MDD [161].

**Table 3:** Amino acid levels in animal models and patients with depression.

Amino Acids (AA)	AA Levels in Animal Models of Depression			AA Plasma Content in Patients with Depression	
**Alanine**	**Direction of Change**	**Biosamples, Animal Model**	**Reference**	**Direction of Change**	**Reference**
	Decreased	PFC, rat, learned helpfulness model	Zhou et al., 2017 [162]	Increased	Mitani et al., 2006 [163]
	Decreased	Feces, rat, chronic unpredictable mild stress model	Jianguo et al., 2019 [158]		
	Decreased Β-alanine	Mononuclear blood cells, rat, chronic unpredictable mild stress model	Li et al., 2014 [164]		
**Arginine**	Decreased	Urine, rat, olfactory bulbectomy model	Zhou et al., 2019 [165]		
**Asparagine**	Abnormalities of arginine metabolism	Plasma, urine, rat, excess fatigue	Zhang et al., 2010 [166]	Decreased	Hess et al., 2017 [160]
**Aspartate**	Increased	Urine, rat, chronic unpredictable mild stress model	Liu et al., 2012 [167]	Decreased	Pu et al., 2020 [156]
**GABA**	Decreased	Mononuclear blood cells, rat, chronic unpredictable mild stress model	Li et al., 2014 [164]	Increased	Steffens et al., 2010 [113]
				Decreased	Lu et al., 2014 [161]
**Glutamic acid**	Decreased	PFC, rat, learned helpfulness model	Zhou et al., 2017 [162]	Decreased	Lu et al., 2014 [161]
**Glutamine**	Decreased	Mononuclear blood cells, rat, chronic unpredictable mild stress model	Li et al., 2014 [164]	Increased	Inoshita et al., 2018 [97]
**Glycine**	Decreased	PFC, rat, learned helpfulness model	Zhou et al., 2017 [162]	Decreased	Pu et al., 2020 [156] Lu et al., 2014 [161]
	Increased	Urine, rat, chronic unpredictable mild stress model	Liu et al., 2012 [167]		
**Isoleucine**	Decreased	Mononuclear blood cells, rat, chronic unpredictable mild stress model	Li et al., 2014 [164]	Increased	Mitani et al., 2006 [163]
	Increased	Urine, rat, chronic unpredictable mild stress model	Liu et al., 2012 [167]		
**Leucine**	Decreased	Feces, rat, chronic unpredictable mild stress model	Jianguo et al., 2019 [158]	Decreased	Baranyi et al., 2016 [157]
**Methionine**	Increased	Urine, rat, olfactory bulbectomy model	Zhou et al., 2019 [165]	Decreased	Baranyi et al., 2016 [157]
**Phenylalanine**	Decreased	PFC, rat, learned helpfulness model	Zhou. et al., 2017 [162]	Decreased	Pu et al., 2020 [156] Baranyi et al., 2016 [157]
**Proline**	Increased	Serum, rat, chronic unpredictable mild stress model	Xiong et al., 2016 [168]	Increased	Steffens et al., 2010 [113]
	Decreased	PFC, rat, learned helpfulness model	Zhou et al., 2017 [162]	–	–
**Serine**	Decreased oxidized proline	Feces, rat, chronic unpredictable mild stress model	Jianguo et al., 2019 [158]	Increased	Hashimoto et al., 2016 [169]
	Decreased	Feces, rat, chronic unpredictable mild stress model	Jianguo et al., 2019 [158]	Decreased	Pu et al., 2020 [156]
**Threonine**	Decreased	Feces, rat, chronic unpredictable mild stress model	Jianguo et al., 2019 [158]	Decreased	Baranyi et al., 2016 [157]
**Tryptophan**	Increased	PFC, rat, olfactory bulbectomy model	Zhou et al., 2019 [170]	Decreased	Pu et al., 2020 [156] Myint et al., 2007 [171]
	Decreased	Serum, rat, chronic unpredictable mild stress model	Xiong et al., 2016 [168]		
**Tyrosine**	Decreased	Feces, rat, chronic unpredictable mild stress model	Jianguo et al., 2019 [158]	Decreased	Islam et al., 2020 [172]
**γ-glutamyl leucine and γ-glutamyl glutamine**	–	–	–	Increased	Hashimoto et al., 2016 [169]

**Table 4:** Antidepressant and anxiolytic effects of AAs in preclinical and clinical settings.

Amino Acids	Type of Study	Effects	Reference
**Alanine**	Rodent model of acute stress	Elicited an anxiolytic-like effects in the elevated plus maze Decreased the concentration of 5-hydroxyindoleacetic acid et al., a major metabolite of serotonin et al., in the hypothalamus Increased carnosine beta-alanyl-L:histidine concentration in the cerebral cortex and hypothalamus Increased brain-derived neurotrophic factor levels in the hippocampus	Murakami et al., 2010 [173]
**Arginine**	Rat model of chronic mild stress-induced depression.	Increased the sucrose preference ratio Increased locomotor activity Increased monoamines Decreased serum cortisol and NO levels Increased BDNF mRNA expression	Dong et al., 2020 [174]
**Glutamine**	Rodent model of immobilization stress	Increased glutamatergic neurotransmission	Son et al., 2016 [175]
**Leucine**	Murine model of inflammation-induced depression	Antidepressant-like effects on behavior Decreased brain kynurenine levels	Walker et al., 2019 [176]
**Methionine**	Comprehensive review of the efficacy of S-adenosyl-L-methionine in MDD	Effective in patients nonresponsive to selective serotonin reuptake inhibitors and serotonin-norepinephrine reuptake inhibitors	De Berardis et al., 2016 [177]
**Serine**	Murine model	D-serine administration significantly reduced immobility in the forced swimming test	Malkesman et al., 2012 [178]
	Srrtg transgenic mice	Elevated brain D-serine levels and reduced proneness towards depression-like behavior	Otte et al., 2013 [179]
**Tryptophan**	Patients	Improved mood	Muszyńska et al., 2015 [180]
**Tyrosine**	Rats fed L-tyrosine-loaded nanoparticles.	Decreased the immobility time in the FST et al., concomitant with restoration of the basal levels of locomotor activity, distance travelled and rearing counts Increased sucrose consumption	Alabsi et al., 2016 [181]

Isoleucine plays a crucial role in the mammalian CNS. Isoleucine is transported across the blood-brain barrier to the brain where it is used as the main amino group donor for GLU synthesis [182]. A study conducted by Baranyi et al. reported reduced isoleucine levels in depressed patients infected with hepatitis C compared to non-depressed HCV-infected patients. The same authors pointed out that isoleucine is a potential immunosuppressant, which is well aligned with the inflammatory hypothesis of depression [183].

TRP is the precursor for 5-HT, 90% of which is produced in the gut and 10% in the brain [184]. Only a small percentage (1–3%) of dietary TRP crosses the blood-brain-barrier into the brain, where it is converted into 5-HT. Today, the conventional view is that the balance between the pathways of TRP metabolism in the body is one of the factors contributing to the body’s predisposition to depression. Patients with higher depression scores may have reduced plasma levels of L-tryptophan [171]. Low blood TRP levels amount to less 5-HT synthesis in the brain. Certain microbial species have been shown to increase plasma TRP levels. As experiments on GF animals show, the GM affects TRP availability in the body, thereby decreasing 5-HT levels in the brain [20]. Plasma TRP levels in GF animals are significantly higher than in conventional animals, most likely because the GM is involved in TRP metabolism. This hypothesis is supported by much evidence from studies of the effects of microbial colonization of GF animals, which induced the expression of many enzymes involved in TRP metabolism [185]. Changes in TRP metabolism were also seen in animals with transplanted microbiota of patients with depression.

The amount of intestinal TRP, which is not transferred to the systemic circulation after absorption, may be locally transformed by different gut microbial species into many catabolites via various metabolic pathways. In the *Firmicutes* phylum, *Clostridium sporogenes,* and *Ruminococcus gnavus* convert TRP into tryptamine, a biogenic amine structurally similar to 5-HT, using tryptophan decarboxylases [111]. Tryptamine-producing *Ruminococcus gnavus* is a common species of the GM found in around 90% of adults [186] and infants [187]. This is all the more interesting since tryptamine has an important role in maintaining gut homeostasis. Endogenous TRP is another well-studied microbiota-related metabolite.

Many gram-positive and gram-negative bacteria produce indole, which led some authors to suggest that indole is an important signaling molecule affecting both bacterial and human physiology [80]. An indolic compound is produced by the enzyme tryptophanase expressed in a large number of microorganisms, including *Escherichia coli*, *Proteus vulgaris*, *Paracolobactrum coliforme*, *Achromobacter liquefaciens*, and *Bacteriodes spp.* and possesses three permeases for TRP transport and produces indole metabolites as by-products of the amino acid metabolism to obtain carbon and nitrogen [79]. Excessive amounts of indole produced by the GM have been shown to increase anxiety-like and depression-like behavior in rats. In a more recent study, indole was linked to the vulnerability of mice exposed to chronic mild stress and interfered with catecholamine biosynthesis in the adrenal medulla [188]. Microbial indole triggered enteroendocrine L-cells to produce glucagon-like peptide 1 (GLP-1), which in turn stimulated colonic vagal afferent activity [189]. The administration of Isatin, an indole derivative seen as a neuroprotective agent, to mice altered the expression of many genes as well as the proteome in their brains [190]. Indole-3-lactic acid, which is another indole derivative produced by lactobacilli and bifidobacteria [191], exerted an enhancing effect on neuron differentiation in PC12 cells via AhR receptors [192]. Indole-3-propionic acid (IPA) is a very powerful antioxidant molecule, produced by *Clostridium sporogenes*, which had gained much attention related to its great potential in protecting the organism against oxidative stress (OS) [193], reducing inflammation [194], and even treating neurodegenerative diseases due to its ability to attenuate endoplasmic reticulum stress, thereby preventing neuronal cell death [195]. *Clostridia*, *Bifidobacteria* and *Bacteroides* spp. metabolize TRP into indole-3-acetic acid (IAA) via multiple pathways, one of which requires a prior transformation of TRP to IPA, and another one including other intermediary molecules such as tryptamine and indole-3-acetaldehyde [196]. IAA is also a plant hormone used by many endophytic bacteria for various purposes [197,198]. The impact of IAA on health has only been addressed in one study where it was associated with cognitive impairment in hemodialysis patients [199]. Indole has many derivatives, whose impact on the CNS is yet to be fully unveiled.

The tryptophan–kynurenine pathway is one of the more diverse pathways of AA metabolism. The products of this pathway encompass both the neuroprotective kynurenic acid and the neurotoxic 3-hydroxykynurenine and quinolinic acid metabolites. Decreased levels of kynurenic acid have been associated with increased neurotoxicity in depression. The heightened levels of quinolinic acid in the frontal cortex are associated with an elevated suicide risk in mood disorder patients [102]. Depression is sometimes explained in terms of an aberrant KYN/TRP pathway [200]. An elevated ratio of plasma KYN/TRP often correlates positively with depression severity in humans [181,201]. A number of meta-analyses reported decreased levels of TRP, kynurenic acid, and kynurenine and increased GLU levels, in MDD patients [97,100,101].

Patients with depression are marked by an increased activity of key enzymes involved in TRP catabolism, tryptophan 2,3-dioxygenase, and indodelamine 2,3-dioxygenase [202]. As a consequence, TRP is metabolized before reaching the brain, which explains why low plasma TRP levels are common in affective disorders [202,203].

D-serine is one of many amino acids exerting antidepressant effects as attested by animal studies conducted in rodent models of depression. [178,179]. The mode of action of D-serine is likened to that of ketamine. [204]. D-serine may be an indicative biomarker of the antidepressant response to ketamine [205].

Leucine also exhibits antidepressant-like effects in mice accompanied by a reduction in brain kynurenine levels [176]. The protective effects of leucine against inflammatory-induced depression are ascribed to its ability to block kynurenine from entering the brain [206].

The main members of the GM that are known to produce AAs are: *Streptococcus* spp., *Staphylococcus aureus*, *Escherichia coli*, *Klebsiella* spp., *Selenomonas ruminantium*, *Megasphaera elsdenii*, *Prevotella* spp., *Bacteroides* spp., and *Clostridium* spp. [207].

Bacteria of the genus *Pseudomonas* possess two genes involved directly in the conversion of TRP into kynurenine, namely KynA and KynB, which encode the enzymes TDO and kynurenine formamidase, respectively [208]. *E. coli* have been shown to produce kynurenic acid from kynurenine due to the enzyme aspartate aminotransferase possessing kynurenine aminotransferase activity [76]. Other enzymes that allow *E. coli* to synthetize quinolonic acid from aspartate are aspartate oxidase and quinolinate synthase [78].

AAs such as glutamine/glutamate, asparagine/aspartate, lysine, serine, threonine, arginine, glycine, histidine and branched-chain AAs might be more readily degraded by gut bacteria [207].

AAs are implicated in almost all the biochemical reactions taking place in the human body. Therefore, AA imbalance is a potential determinant of various diseases, including mental disorders. AAs also play a key role in the metabolic pathways of bacteria, which makes them involved in the interplay between the gut and the brain. Some AA and their metabolites of bacterial origin can be considered as biomarkers of depression in clinical practice.

## 6. SCFAs as Biomarkers of Depression

Some of the most important bacterial metabolites mediating the communication between the gut and the brain are SCFAs. Bacteria produce SCFAs as a byproduct of the fermentation of non-digestible carbohydrates [209]. The resulting end products include acetate, propionate, and butyrate [210] and, to a lesser extent, iso-butyrate, valerate and iso-valerate [187]. The lipophilic nature of SCFAs allows them to easily reach the brain by crossing the blood-brain-barrier, where they interact with neurons. Conversely, the salubrious properties of SCFAs could emerge from their strengthening properties of the intestinal barrier, the major crossing point of molecules and nutrients from the bloodstream to the brain. Although the exact mechanisms of action of SCFAs are not completely clear, their therapeutic potential for treating neurological and neurodegenerative disorders is widely supported by animal studies [211]. SCFAs activate G-protein coupled receptors located on endocrine and immune cells, kidneys, blood vessels, and nerve cells. SCFAs exhibit anti-inflammatory properties, and low SCFA levels characteristic of a “dysbiotic” GM could be one of the causes of inflammation in depression

Many animal studies have confirmed the antidepressant effects of SCFAs, directly or indirectly. For instance, the transplantation of fecal microbiota from patients with depression to sterile rats made them anxious and increased fecal SCFA levels compared to rats receiving donor microbiota from healthy individuals [49]. In an animal model of mania, the administration of sodium butyrate, a major SCFA, restored normal levels of activity and mitochondrial function in the PFC, hippocampus, striatum, and amygdala [212]. Sodium butyrate also abrogated depressive-like and mania-like behavior in rats [213].

Acetic, propionic, and caproic acids have been shown to partly contribute to the origin of symptoms of depression through the MGBA. Acetate exerts protective activity against enteropathogenic infections and fortifies the gut barrier. Lower levels of acetate observed in patients with depression translate into a decrease in butyric acid. Propionate dampens the innate immune cell response to bacteria, and may also be involved in keeping intestinal permeability in check [127,214]. Lower levels may contribute to dysbiosis and neuroinflammation in the CNS, which are known risk factors for depression. In the study by El-Ansary et al. the administration of propionate to animals was followed by changes in their phospholipid and acylcarnitine profiles. Besides its direct effect on the brain, propionate modulates the secretion of 5-HT in the gastrointestinal system and causes a decline in brain 5-HT and dopamine levels [215,216]. Oral administration of propionate induced lipid peroxidation in rodents and led to a decreased activity of glutathione, glutathione peroxidase and catalase [217]. In the study of Skonieczna-Zydecka, acetate and propionate levels correlated inversely with the symptoms of depression, as evaluated with the Beck depression inventory. Fecal levels of isocaproic acid were significantly lower in Polish women with depression [104].

According to a study of the effects of butyrate on behavior, memory, and levels of neurotrophic factors in a rat model of chronic mild stress, butyrate functions as an antidepressant [218]. Aside from regulating the levels of neurotrophic factors, butyric acid typically inhibits histone deacetylation and prevents hippocampal microglia activation. Bacteria-derived butyrate has been shown to modulate the synthesis of dopamine, noradrenaline, and adrenaline by altering the expression of the tyrosine hydroxylase-encoding gene [219].

Isovaleric acid is another SCFA that was found to be correlated with the symptoms of depression and blood levels of cortisol [54]. Isovaleric acid interferes with the release of neurotransmitters into the synaptic cleft, which implies a causal relationship between it and depression. For instance, isovaleric acid levels in the colon can directly impact the functioning of the hypothalamus in the brain [54]. Understandably, bacteria capable of altering the levels of valeric acid in the gut are an interesting target for treating depression. Valeric acid, due to its structural similarity to GABA, acts as an inverse agonist of the adenosine A1 receptor in the brain, which is known for its role in the regulation of neurotransmitter release [54].

The genera of enteric bacteria that contribute to acetic acid production are *Akkermansia muciniphila, Bacteroides* spp., *Bifidobacterium* spp., *Prevotella* spp., *Ruminococcus* spp., *Blautia hydrogentrophica,* and *Streptococcus* spp. [81,84].

The organism’s supply of butyrate is exclusively derived from butyrate producing bacteria, which include: *Coprococcus comes*, *Coprococcus eutactus*, *Anaerostipes* spp., *Coprococcus catus*, *Eubacterium rectale*, *Eubacterium hallii*, *Eubacterium rectale*, *Roseburia inulinivorans*, *Roseburia intestinalis*, *Clostridium symbiosum*, and *Faecalibacterium prausnitzii* [81,85,86].

The bacterial genera and species involved in propionic acid production are the following: *Bacteroides* spp., *Phascolarctobacterium succinatutens*, *Dialister* spp., *Veillonella* spp., *Megasphaera elsdenii*, *Coprococcus catus*, *Salmonella* spp., *Roseburia inulinivorans*, *Ruminococcus obeum*, *Dalister succinatiphilus*, *Eubacterium* spp. (e.g., *E. halli*), *Roseburia* spp., *Veillonella* spp., *Akkermansia muciniphilia*, *Clostridium* spp., and *Ruminococcus* spp. [220].

The production of valeric acid was associated with the genera *Acidobacteria*, *Paracoccus denitrificans* [221].

Faecal proteolytic bacteria *Propionibacteriurn* spp*., Clostridium* spp., *Streptococcus* spp., *Bacillus* spp. and *Staphylococcus* spp. [222] can extract iso-butyric and iso-valeric acid from proteins.

Since SCFAs are bacterial metabolites, their levels in the body completely depend on the GM composition. Existing studies that reveal the potential of SCFA as biomarkers of depression confirm the correlation of the gut bacteria with the pathogenesis of the disease. This may potentially expand the diagnostic criteria for depressive disorders in clinical practice.

## 7. Other Bacterial Metabolites as Biomarkers of Depression

Since depression was formulated as a mental illness, many studies have focused on identifying potential blood-based biomarkers [223,224]. MDD patients were characterized by higher levels of asymmetric dimethylarginine, tyramine, 2-hydroxybutyric acid, phosphatidylcholine (32:1), and taurochenodesoxycholic acid and lower levels of l-acetylcarnitine, creatinine, linoleic acid, pyruvic acid, palmitoleic acid, oleic acid, myo-inositol, dodecanoic acid, hypoxanthine, palmitic acid, taurine, 25-hydroxyvitamin D [225].

A recent study raised the question of the relevance of vitamin B intake in the light of the negative correlation between it and the symptoms of depression [226,227]. Interestingly, gut bacteria are an important source of B-vitamins, namely, niacin (B-3), biotin (B-7), folate (B-9), and pyroxidine (B-6) [228,229]. Biotin and niacin, in particular, participate in immunomodulation, and deficiency of these vitamins could be one of the reasons of gut and systemic inflammation [230]. Folate levels in the serum of patients with depression are lower than those of controls [105] and may be associated with symptom severity [231] and responsiveness to antidepressant treatment [232]. Folic acid is another product of bacterial metabolism that correlates with symptoms of depression [233]. Folate deficiency is diagnosed in one in three patients with depression. The main folate-producing bacteria belong to species of *Lactobacillus* and *Bifidobacterium* [87]. Pyroxidine, an essential cofactor for many enzymes participating in the kynurenine pathway, is altered in patients with depression [171]. *Lactobacillus* and *Bifidobacterium*, as well as other gut bacteria, synthesize B-group vitamins and vitamin K as part of their core metabolic pathways in the large intestine, and humans rely heavily on the GM as a source of these vitamins [88].

Polyunsaturated fatty acids (PUFAs) are involved in the structural and functional regulation of neurons, glial cells and endothelial cells in the brain, which makes them strongly linked to depression. A recent study has linked inflammation to PUFAs, more specifically with their metabolites [234]. Druart et al. demonstrated the GM’s ability to transform dietary PUFAs into other molecules, pointing out that these metabolites could be of bacterial origin [235]. A comparison of the ratio of PUFA-derived metabolites and the expression of fatty acid desaturases between GF and conventionalized mice revealed higher concentrations of PUFA-derived metabolites in the colonic contents of conventionalized mice. Omega-3 PUFAs are known to modulate the GM composition [236]. Mice fed an omega-3-enriched diet exhibited significantly enhanced cognition, and attenuated HPA-axis activation and inflammation, as well as an improved intestinal epithelial integrity, possibly due to a higher abundance of bifidobacteria [237].

N-acetyl aspartate (NAA), a marker of neuron vitality, was found to be decreased in the prefrontal cortex (PFC) and hippocampus of MDD patients [238,239]. Oral administration of *L. rhamnosus* to mice increased NAA, Glx and GABA levels in the PFC and the hippocampus [240], implicating antidepressive potential of bacterium.

Fatty acids, through their effects on cell membrane structure, biological stress, and inflammatory responses, may be involved in depression [241]. Fatty acid synthesis was among the top-ranked metabolic pathways. The levels of five fatty acids and l-acetylcarnitine were significantly decreased in the blood of MDD patients compared with controls. l-acetylcarnitine plays a pivotal role in the transport of fatty acids into the mitochondria for beta-oxidation, and l-acetylcarnitine supplementation was reported to have antidepressive effects [242]. The blood of patients with depression was also marked by higher levels of taurochenodesoxycholic acid, a bile acid formed from taurine in the liver [156]. This was confirmed in animal models of depression, which reported increased levels of taurochenodesoxycholic acid levels and decreased taurine levels in the liver [243,244], further emphasizing the link between primary bile acid synthesis and MDD. Bile acids are synthesized from cholesterol in the liver and further metabolized by the GM into secondary bile acids [245]. 25-Hydroxyvitamin D is the primary form of vitamin D in the human body. A deficiency in vitamin D has been associated with higher rates of suicide and a proinflammatory profile [246,247].

There are also several gut hormones, including NPY, GLP-1, cholecystokinin (CCK), and ghrelin, that are known to play important roles in mood disorders such as anxiety and depression. The neuropeptide Y family (NPY), including NPY, PYY, and pancreatic polypeptide (PP), are all involved in stress-related disorders, neuroprotection, neuroinflammation, and neurogenesis. Some of those peptides, namely NPY and PP, act by activating Y4 receptors, which are involved in the control of a wide range of behavioral processes [248].

GLP-1 is best known for its role in glucose-dependent insulin secretion. GLP-1 also responds to stress through the activation of the GLP-1 receptor [249]. Another regulator of the stress response is ghrelin, with adipogenic and orexigenic effects also identified as a regulator anxiety and depression [250].

Although the precise pathways mediating the GM communication with hormones have not yet been deciphered, growing pieces of evidence now suggest that microbiota is a key factor involved in the metabolism of hormones, and host hormones greatly influence the microbiota. One example included the reduction in leptin plasma levels following treatment with antibiotics [251]. The ghrelin levels are decreased when the GM composition is altered. When fasting, ghrelin level increased and prebiotic supplementation resulted in lower prospective food consumption and more feeling of fullness. Microbes may also regulate hormone secretion in an indirect way via their influence on the adrenal cortex and inflammatory response, all of which are closely related to the CNS and the GIT. For instance, certain bacterial components and metabolites promote the release of gut hormones from the EECs. Lipopolysaccharides (LPS) bind to Toll-like receptor 4 expressed in L-cells and triggers the secretion of GLP-1 in mice [252]. Gut hormones, on their part, signal ongoing processes to the microbes forcing them to act accordingly. A recent evolutionary-oriented study hypothesized that a variety of hormone metabolism related enzymes might have evolved from bacterial genes [253,254].

Depression is connected with increased oxidative stress [255]. In this regard, the metabolites that somehow have an effect on OS in the body are good markers of depressive disorders. For instance, the study of Zheng et al. revealed increased levels of uric and azelaic acids in the urine [256]. Uric acid is formed in response to increased oxidative stress. Azelaic acid can have an antioxidant effect. The increased production of these compounds indirectly indicates a link between oxidative stress and depression. It is interesting to note that men and women with depression have different markers of OS. While women exhibited high levels of sorbitol and azelaic acid levels, in men, OS was associated with a decrease in cysteine and 1-methylinosine [257].

Glutathione (GSH) is the most abundant antioxidant in human tissue and an important indicator of antioxidant capacity and OS. According to a post-mortem study, the levels of reduced, oxidized, and total GSH are significantly lower in the PFC of MDD patients [106]. In the study of Eren et al. the induction of depressive-like behavior in Wistar rats resulted in a decrease in GSH and gluthatione peroxidase (GPx) levels and a rise in lipid peroxidation and nitric oxide in the cortex and medulla of the brain [258]. Comparative studies of GPx levels between people with depression and controls have yielded conflicting results. GPx levels were decreased [259,260], increased [261], or even unaltered [262] compared with healthy control groups. It is possible that the lack of uniform results between the studies is due to differences in the used methodology and sample selection bias. In bacteria, besides its role in maintaining the proper oxidation state of protein thiol, GSH serves a protective role against low pH, chlorine compounds, oxidative and osmotic stress. The production of GSH among prokaryotes is limited to cyanobacteria and proteobacteria such as *E. coli* and *Listeria monocytogenes*, as well as gram-positive bacteria such as enterococci and streptococci [89,90,91]. GSH is from γ-glutamylcysteine by gluthatione synthetase. The favorable effects of probiotics on GSH levels (increasing synthesis) might be due to enhanced glutamate–cysteine ligase activity [250].

The immune system plays a crucial role in depression pathogenesis. A depressed patient presents immune dysregulation and chronic inflammation [4]. The cytokines, chemokines, endocrine messengers, and microbial products can infiltrate the blood and lymphatic systems or influence neural messages carried by the vagal and spinal afferent neurons, thereby affecting the regulation of the activity of the HPA and neuroinflammation.

Chronic inflammation and microglia activation are common features in depression [263,264]. Immunomodulation by bacteria is the important mechanism of CNS regulation. Many bacterial surface molecules, such as LPS, lipoproteins, and flagellins activate macrophages, neutrophils, and dendritic cells. Once activated, these cells produce proinflammatory interleukins IL-1a, IL-1b, TNFa, and IL-6 [265], which once carried over to the brain; interact with neuronal receptors and glial cells, in particular, turning on their “activation mode” and altering their properties [264]. The patients with depression have higher LPS levels and their immune system is far more active [266]. Some components of microbial cell walls facilitating adhesion to epithelial cells can trigger production and secretion of compounds capable of modulating neuronal signaling [267]. The GABA, acetylcholine, and norepinephrine, which can be produced by bacteria, exhibit immunomodulatory properties.

The identification of biomarkers associated with the GM may potentially expand diagnostic assessment of depressive disorders in clinical practice. The knowledge about the composition and metabolite potential of the GM may be applied as an additional diagnostic criterion as well as contribute to the assessment of disease severity. Together, they represent a future diagnostic tool for assessing the development of depressive disorders.

## 8. The Therapeutic Potential of Functional Foods and Probiotics

Today, diet remains one of the most effective measures that can be taken to restore the microbial balance in the gut [268] and alleviate the symptoms of depression. Given that diet has a profound effect on the composition and functional capacity of the GM, the salubrious potential of dietary intervention is no surprise. Foods rich in fiber and complex sugars act as a source of energy for beneficial bacteria, at the forefront of which are butyrate-producing bacteria. Fats and simple sugars, which are prominently consumed in abundance in the Western diet, should be avoided lest to nurture “bad” microorganisms known for their production of neurotropic metabolites [269]. Animal-derived protein and saturated fats are associated with *Bacteroides* abundance, while *Prevotella* are associated with carbohydrates and simple sugars [270]. A reduction in total carbohydrate in the diet reduces the butyrate-producing *Roseburia/Eubacteriumrectale* group [271]. High-sucrose feeding significantly decreases the abundance of *Bacteroidetes* in mice, while increasing the abundance of *Proteobacteria*, *Firmicutes*, and pathogenic *Helicobacteraceae* [272]. Vegetable-based diets can increase SCFAs, accompanied by elevated *Prevotella* and some fiber-degrading *Firmicutes* [273]. Increasing dietary fat alters the GM composition [219,236], possibly via the stimulation of bile and its modulation into secondary bile acid products [274].

The correlative studies of healthy adults showed that a lower incidence of depression occurs when using “healthy” dietary patterns with an abundance of vegetables, fruits, as well as moderate amounts of dairy, eggs, and fish, and unsaturated fats [275,276,277]. A 10-y longitudinal study in France showed an association between depression incidence and poor diet, but found that there was most likely reverse causality, with depression increasing the risk of poor eating behaviors [278].

A diet poor in PUFAs is more than likely to contribute to the development of depression [234]. Moreover, patients with depression display lower serum levels of omega-3 and of omega-6 PUFAs, both used as building blocks by nerve cells [279]. Insufficient intake of fish, meat and nuts offers often a valid explanation for PUFA deficiency. Therefore, dietary supplementation with foods rich with omega-3 and omega-6 PUFAs can efficiently ameliorate depression symptoms and improve cognitive function.

By the same token, replacing inflammatory foods, such as sugar, gluten, artificial trans fats, chemical additives, taste enhancers, and preservatives with anti-inflammatory foods, such as berries, avocado, broccoli, olive oil, cocoa, and green tea, can be a huge step towards mitigating the heightened inflammatory response of the immune system [280]. Fungal glycans, phytosterols and flavonoids also have immunomodulating properties. There is enough evidence showing that diet is a powerful modulator of the immune response and a determinant of social behavior in patients with depression. For example, luteolin, a natural flavonoid, inhibits the release of proinflammatory IL-6 from mast cells [281].

Functional foods hold great promise as enhancers of aberrant behavior and cognition in people with depression. A functional diet consumed on a daily basis, whether consisting of natural or synthetic nutrients, can primarily normalize the intestinal microecology and secondarily recover vital physiological functions and biochemical reactions. Functional foods fulfill both a protective and a preventive function. There is a wide variety of functional foods with potential curative effects with regard to depression. These are foods rich in fiber (prebiotics), probiotics (bifidobacteria and lactobacilli), antioxidants and vitamins (A, E, C, and group B), mineral elements (calcium, microelements (iron, zinc, fluorine, selenium, etc.), PUFAs, plant sterols, conjugated linoleic acid isomers, structured lipids, sphingolipids, polysaccharides and secondary plant-derived compounds (flavonoids, polyphenols, carotenes, licopene), taurine, co-enzyme Q10, and carnitine.

Strains of lactobacilli and bifidobacteria can be selected based on their genetic and genomic differences favoring those that are more likely to impart health benefits to the nervous system in humans [282]. Bifidobacteria, in particular, are known for their ability to counter inflammation [283]. Such elaborately chosen strains can be taken as live food supplements. In the last decades, the discovery of unique strains of lactobacillus and bifiobacteria capable of improving symptoms of psychiatric diseases and even alter behavior led to a paradigm shift in the field of psychiatry [284,285]. These bacteria were named ‘psychobiotics’ and were defined as live bacteria that can benefit mental health. Psychobiotics were proposed as a viable alternative to standard pharmacological treatment, which is often discredited for its infamous adverse effects and controversial effectiveness. Psychobiotics secrete a wide range of signaling molecules that operate via distinct pathways to exert their effects, be it antidepressive, immunomodulatory, or modulation of neurotransmission. Different strains of psychobiotics were tested within both preclinical and clinical setups for their potential to treat of depression. While some studies failed to reproduce the animal results in humans, others yielded robust antidepressant effects coupled with a shift in the *Bacteroidetes*:*Firmicutes* ratio and increased the abundance of bifidobacteria [286]. Some psychobiotics were recommended as adjuvants for anti-depressant therapy [287,288]. Since diet largely determines the composition of the GM, it is an important variable in the connection between the gut and nervous system diseases.

Because diet has a greater impact on the composition and function of the GM, it is likely that changes in diet (whole diet and individual components of diet) may affect depression. Currently, research in this area is limited and mainly conducted on rodents. Dietary models for positive effects on mental health will focus on maintaining the growth of beneficial commensal microbiota, reducing the growth of pathogenic microbiota, and influencing intestinal barrier permeability and inflammation. However, it is important to remember that diet will affect the collective function and characteristics of the GM that interact with the host. Similar functions can be carried out by different microbiota structures and the same functional outcome could occur with different changes in microbiota. Therefore, the type and strength of the effect of diet on the GM will be determined by the existing composition and function of the microbiota. Research needs to include examination of the GM function using metabolomics and/or metagenomics techniques.

A healthy diet during the depression therapy, along with the application of probiotics and psychobiotics, may potentially improve the course of the disease and contribute to the progress of treatment.

## 9. The Utility of Reference Gene Catalogs for Understanding the GM

The GM developed in the process of evolution the necessary machinery in the form of specialized enzymes that enabled it to produce various active compounds. Overall, the functional potential of the GM depends on the diversity and relative abundance of bacterial genes. As the cost and accuracy of high-throughput sequencing evolved to meet the needs of scientists, the analysis of collective bacterial genomes known as metagenomes, have become an irreplaceable and reliable tool in GM research [289]. Whole metagenome sequencing allows us to search for specific bacterial genes and rebuild the functional capacity of the sampled microbiota. However, such analyses can yield meaningful results only when the right targeted genes are built into the reference gene catalog of the search tool. Table 5 provides an example of a gene catalog comprising the key bacterial enzymes relevant to depression.

There are many ways to functionally annotate a metagenome. The easiest way is to use the RefSeq database, which is one of the richest databases in homologous sequences [290]. While such an approach may generate high-resolution results, it still produces a large number of false-positive results due to a poor representation of certain bacterial metabolic pathways. For instance, to produce dopamine, gut bacteria use tyrosine decarboxylase rather than dopa decarboxylase [291]. The second way is to use databases of metabolic pathways such as KEGG [292] or MetaCyc [293]. This approach is more reliable since, as in the case of KEGG and MetaCyc, such databases are constantly updated. Although such a detailed analysis may be applicable for characterizing the functional potential of metagenomes, the results remain of little diagnostic value. The third way consists of selecting the genes of interest and by creating a customized gene catalog. Gene catalogs consist of consensus sequences of genes that are obtained by consulting the main research databases such as Pubmed, databases such as SwissProt, InterPro, KEGG, MetaCyc, etc. The genes are selected in conformance with the envisaged purpose of the gene catalog, such as to determine the neuromodulatory potential of the metagenome. Reference gene catalogs not only allow to detect the targeted genes but also evaluate the relative abundance of these genes in a metagenome sample. Several variants of reference catalogs of genes involved in the synthesis of neuroactive compounds currently exist. The first catalog was developed by Kovtun et al. [294]. This gene catalog was designed to characterize the core metagenomic neuromodulatory potential of the human’s GM in the norm. The second variant was extended to include genes involved in the production of compounds relevant to autism spectrum disorders [24]. The gene catalog was proven effective for revealing significant changes in the metagenomic signature of the GM of children with ASD from the Moscow region [24]. Presently, more genes are being added to the gene catalog, thereby redirecting its purpose to various neuropsychiatric disorders. The complete composition of the catalog at the moment of writing of this review is provided in Table 5. Another gene catalog aimed at characterizing the neuroactive potential of the human GM was created by Valles-Colomer et al. [53]. The gene catalog was successfully utilized by the authors to search for associations between the GM and depression and schizophrenia [53,295]. 

## 10. Conclusions

Depression is multifactorial disease and today, different theories and hypotheses aspiring to explain the etiology of depressive disturbance. They range from dysfunction of neurotransmitter systems to neuroinflammation, metabolic abnormality, deficit of neurotrophic factors, and others. Although a large number of depression biomarkers have been identified, an effective panel of markers is still lacking in clinical practice. Despite the knowledge about the participation of the GM in the bidirectional communication between the gut and the brain and its role in the development of various neuropsychiatric disorders the study of the influence of the GM on depressive disorders is currently in its infancy. The data presented in this review promotes the understanding of the role of the GM in the pathogenesis of depression.

The purpose of this review was to discuss biomarkers of depression correlating with the GM, which included the neurotransmitters serotonin, norepinephrine and/or dopamine, GABA and glutamate; SCFAs; amino acids and other metabolites with neuro- and immunomodulation. The relationship between these biomarkers and depression has been shown in animal models and humans. The observed changes in the composition of the GM of patients with depression provide to the development of new strategies to depression therapy through the impact on the GM. In this review, we described the therapeutic potential of the functional foods and psychobiotics and their antidepressant effects. These strategies provide an effective alternative for the treatment of mental disorders.

The use of the GM biomarkers, reflecting the neuromodulatory, immunomodulatory and antioxidant statuses of the host organism, in the analysis of metagenomic data from patients with neuropsychiatric diseases, is gaining currency. Creating gene catalogs containing functional genes will allow purposeful analyze of the metagenomic data. Defining the metagenomic signature in the norm is crucial for singling out the genes with a diagnostic potential in the context of depression. Bacterial genes encoding enzymes involved in the metabolism of biomarkers described in this review most likely determine the neuro- and immunomodulatory potential of the microbiomes that contribute to depressive manifestations, and can be used to identify the metabolic signature of the GM of patients with depression.

Further progress in our practical understanding of the role of the GM in depression will depend greatly on correct planning of future metagenomic studies. These studies need to be uniformly carried out by criteria of the formation of patient cohorts, choosing definitive biochemical and genetic biomarkers, and using new bioinformatics approaches for deeper analysis. The new knowledge about the GM will form the basis for the development of fundamentally new methods of treatment and diagnosis of depression in clinical practice.

## Figures and Tables

**Table 1:** Gut bacteria and their enzymes that are involved in the production of metabolites relevant to depression.

Metabolite	Bacterial Enzymes Involved in the Production of the Metabolite	Microbial Genera and Species Involved in the Production of the Metabolite	Reference
**Serotonin**	Aromatic amino acid decarboxylase	*Pseudomonas putida*, *Bacillus subtilis, Staphylococcus aureus, Escherichia coli, Morganella morganii, Klebsiella pneumonia, Lactobacillus helveticus*, *Lactobacillus plantarum, Enterococcus faecalis, Lactococcus lactis* subsp. *cremoris, L. lactis* subsp. *lactis*	Shishov et al., 2009 [64] Özoğul et al., 2004 [65] Özoğul et al., 2012 [66] O’Mahony et al., 2015 [67] Dinan et al., 2015 [68]
**Dopamine**	DOPA decarboxylase	*B. subtilis, B. mycoides, B. cereus, S. aureus, P. vulgaris, S. marcescens, E. coli , M. morganii, K. pneumonia*, *Hafnia alvei*, *L. helveticus* NK-1, *L. delbrueckii* subsp. *bulgaricus*	Tsavkelova et al., 2000 [69] Özoğul et al., 2004 [65] Shishov et al., 2009 [64]
**Noradrenaline**	Dopamine β-hydroxylase	*Bacillus mycoides*, *B. subtilis, Proteus vulgaris, Serratia marcescens, E. coli*	Tsavkelova et al., 2000 [69] Shishov et al., 2009 [64]
**GABA**	Glutamate decarboxylase	*Bifidobacterium* spp., *Lactobacillus* spp., *Bacteriodes* spp., *Alistipes* spp., *Parabacteroides* spp.*, Escherichia coli*	Yunes et al., 2016 [70] Strandwitz et al., 2019 [71]
**L-glutamate**	Glutaminase	*Corynebacterium* spp.*, L. plantarum. L. paracasei, Lactococcus lactis*, *L. rhamnosus*, *Brevibacterium* spp., *Ruminococcus* spp.	Stromeck et al., 2011 [72] Lin et al., 2013 [73] Liu et al., 2017 [74]
**Phenylalanine**	Horismate mutase/prephenate dehydratase (PheA)	*Corynebacterium glutamicum Escherichia coli*	Ikeda et al., 2006 [75]
**Tryptophan**	Tryptophan synthetase	*Clostridium* spp., *Pseudomonas* spp., *Bifidobacterium* spp., *Streptomyces* spp., *C. glutamicum,* *E. coli,* *S. typhimurium*	Ikeda et al., 2006 [75]
**Kynurenic acid**	Kynurenine aminotransferase	*E. coli, Pseudomonas* spp.	Han et al., 2001 [76] Sofia et al., 2018 [77]
**Quinolonic acid**	3-hydroxyanthranilic acid oxygenase quinolinate synthase	*E. coli*	Katoh et al., 2006 [78]
**Indole**	Typtophanase	*Clostridium* spp*. E. coli,* *Proteus vulgaris, Paracolobactrum coliforme, Achromobacter liquefaciens*, *Klebsiella* *oxytoca, Providencia stuartii, Citrobacter koseri, M. morganii, Haemophilus influenza, Stigmatella aurantiaca,* *Lactobacillus, B. longum, Bacteriodes fragilis, Parabacteroides distasonis,* *Eubacterium* *hallii*	Yanofsky et al., 1991 [79] Lee et al., 2010 [80]
**Propionic acid**	Propionaldehyde dehydrogenase methylmalonyl-CoA decarboxylase	*Bacteroides* spp., *Phascolarctobacterium succinatutens*, *Dialister* spp., *Veillonella* spp., *Megasphaera elsdenii*, *Coprococcus catus, Salmonella* spp., *Roseburia inulinivorans, Ruminococcus obeum, Dalister succinatiphilus, Eubacterium* spp. (e.g., *E. halli*), *Roseburia* spp., *Veillonella* spp., *Akkermansia muciniphilia*, *Clostridium* spp., *Ruminococcus*	MacFabe et al., 2011 [37] Louis et al., 2014 [81] Ara Koh et al., 2016 [82] Markowiak-Kopec et al., 2020 [83]
**Acetic acid**	Phosphotransacetylase	*Akkermansia muciniphila, Bacteroides* spp., *Bifidobacterium* spp., *Prevotella* spp., *Ruminococcus* spp., *Blautia hydrogentrophica* and *Streptococcus* spp.	Louis et al., 2014 [81] Rey et al., 2010 [84]
**Butyric acid**	Butyrate kinase	*Coprococcus comes, Coprococcus eutactus, Anaerostipes* spp.*, Coprococcus catus, Eubacterium rectale, Eubacterium hallii, Eubacterium rectale, Roseburia inulinivorans, Roseburia intestinalis, Clostridium symbiosum* and *Faecalibacterium prausnitzii*	Miquel et al., 2013 [85] Duncan et al., 2002 [86] Louis et al., 2014 [81]
**Folate**	Dihydrofolate synthase	*Lactobacillus*, *Bifidobacterium*	Rossi et al., 2011 [87]
**Pyroxidine**	Pyridoxine 5′-phosphate oxidase	*Lactobacillus*, *Bifidobacterium*	Gu et al., 2016 [88]
**Total glutathione**	Glutathione synthetase	*Lactobacillus* spp.*, L. lactis,* *E. coli, Listeria monocytogenes,* enterococci and *streptococcus*	Fahey et al., 1991 [89] Newton et al., 1996 [90] Gopal et al., 2005 [91]

**Table 2:** Altered metabolites in patients with depression.

Metabolites	Direction of Change in Patient’s		
	Direction of Change	Biosamples	Reference
**Serotonin**	Decreased	Plasma	Saldanha et al., 2011 [92] Pan et al., 2018 [93]
**Dopamine**	Increased	Blood, plasma	Zheng et al., 2016 [94]
	Decreased	Peripheral blood cells	Zhao et al., 2015 [95]
**Noradrenaline**	Increased	Blood plasma, urine	Valles-Colomer et al., 2019 [53]
**GABA**	Decreased	Blood serum	Madeira et al., 2018 [96]
**L-glutamate**	Increased	Cerebrospinal fluid	Inoshita et al., 2018 [97]
	Increased	Peripheral blood	Zheng et al., 2010 [98] Shao et al., 2013 [99]
**Phenylalanine**	Decreased	Urine	Ogawa et al., 2014 [100]
**Tryptophan**	Decreased	Blood plasma	Ogyu et al., 2018 [101]
**Kynurenic acid**	Decreased	Blood plasma and serum	Bryleva et al., 2017 [102]
**Quinolonic acid**	Increased	Frontal cortex	Jaglin et al., 2018 [103]
**Indole**	Increased	Stool	Skonieczna-Zydecka et al., 2018 [104]
**Propionic acid**	Decreased	Stool	Skonieczna-Zydecka et al., 2018 [104]
**Acetic acid**	Decreased	Stool	Skonieczna-Zydecka et al., 2018 [104]
**Butyric acid**	Decreased	Stool	Bottiglieri et al., 2000 [105]
**Folate**	Decreased	Serum	Gawryluk et al., 2011 [106]
**Total glutathione**	Decreased	PFC	Saldanha et al., 2011 [92]

**Table 5:** Gene catalog of the key bacterial enzymes relevant to depression.

Functions	Enzymes	Functions	Enzymes
Synthesis of serotonin, dopamine and norepinephrine	Dopa decarboxylase	Synthesis of butyrate	Butyrate kinase
Synthesis of GABA	Glutamate decarboxylase	Formation of butyric acid	Butyryl-CoA dehydrogenase
Transportation of GABA	Gamma-aminobutyrate antiporter	Formation of propionic acid	Lactoyl-CoA dehydratase
Degradation of GABA	4-aminobutyrate aminotransferase (gabT)		Propionaldehyde dehydrogenase
	4-aminobutyrate aminotransferase (puuE)		Methylmalonyl-CoA decarboxylase
	Glycine amidinotransferase	Conjugation of linoleic acid	Linoleic acid isomerase
Synthesis of histamine	Histidine decarboxylase	Synthesis of spermidine	Spermidine synthase
Degradation of serotonin for melatonin synthesis	Serotonin N-acetyltransferase	Synthesis of tyramine and dopamine	Tyrosine decarboxylase
Synthesis of melatonin	Acetylserotonin O-methyltransferase	Synthesis of isovaleric acid (KADH pathway)	2-oxoisovalerate dehydrogenase alpha
Formation of nitric oxide	Nitric oxide synthase		2-oxoisovalerate dehydrogenase beta
Degradation of nitric oxide	Nitric oxide dioxygenase		Dihydrolipoyl dehydrogenase
	Nitric oxide reductase norB	Synthesis of isovaleric acid (KADC pathway)	Aldehyde dehydrogenase
	Nitric oxide reductase norC		Pyruvate decarboxylase
Synthesis of catecholamines	Aromatic amino acid hydroxylases	Synthesis of inositol	Myo-inositol-1(or 4)-monophosphatase
Degradation of serotonin, dopamine and norepinephrine	Monoamine oxidase		Myo-inositol-1-phosphate synthase
Formation of acetic acid	Phosphotransacetylase	Degradation of inositol	Myo-inositol 2-dehydrogenase
Degradation of γ-hydroxybutyric acid	4-hydroxybutyrate dehydrogenase	Degradation of glutathione	Glutathione S-transferase
Synthesis of glutamate II	Glutamate synthase gltB		Glutathione reductase
	Glutamate synthase gltD		Gamma-glutamyltranspeptidase
Degradation of glutamate II	Glutamate mutase glmS	Degradation of histidine	Histidine ammonia-lyase
	Glutamate mutase glmE	Synthesis of 4-etylphenol	Vinylphenol reductase
	Methylaspartate ammonia-lyase	Synthesis of indole from tryptophane	Tryptophanase
Synthesis of p-cresol	4-hydroxyphenylacetate decarboxylase	Synthesis of prephenate	Chorismate mutase
Degradation of p-cresol	4-cresol dehydrogenase	Synthesis of 4-hydroxyphenylpyruvate	Prephenate dehydrogenase
	Protocatechuate 3,4-dioxygenase pcaG	Transportation of tyrosine	Tyrosine-specific transport protein
	Protocatechuate 3,4-dioxygenase pcaH	Synthesis of tyrosine	Tyrosine aminotransferase
Synthesis of creatinine	Creatinine amidohydrolase	Synthesis of phenylalanine	Phenylalanine aminotransferase
Formation of D-lactic acid	D-lactate dehydrogenase	Transportation of phenylalanine	Phenylalanine-specific permease
Synthesis of glutathione	Glutathione synthetase, gshB	Synthesis of tryptophan	Tryptophan synthetase, subunit alpha
	Glutathione synthetase, gshAB		Tryptophan synthetase, subunit beta
Transportation of tryptophan	Tryptophan-specific transport protein		
	Tryptophan permease		
Antioxidants	Superoxide dismutase, [Mn]		
	Superoxide dismutase, [Fe]		
	Superoxide dismutase, [Cu-Zn]		
	Catalase		
	Glutathione peroxidase		
